# Copper Phthalocyanine Chemiresistors as Industrial NO_2_ Alarms

**DOI:** 10.3390/s25092955

**Published:** 2025-05-07

**Authors:** Hadi AlQahtani, Mohammad Alshammari, Amjad M. Kamal, Martin Grell

**Affiliations:** 1 Department of Physics and Astronomy, College of Science, King Saud University, Riyadh 11451, Saudi Arabiaamazhar@ksu.edu.sa (A.M.K.); 2 Faculty of Science and Technology, University of Chuka, Chuka P.O. Box 109-60400, Kenya; mgrell@chuka.ac.ke; 3 Llyfrgell Bangor, Ffordd Gwynedd, Bangor LL57 1DT, UK

**Keywords:** nitrogen dioxide, sensor, chemiresistor, copper phthalocyanine, fertiliser

## Abstract

We present a chemiresistor sensor for NO_2_ leaks. The sensor uses the organometallic semiconductor copper(II)phthalocyanine (CuPc), and is more easily manufactured and characterised than previously described organic transistor gas sensors. Resistance R is high but within the range of modern voltage buffers. The chemiresistor weakly responds to several gases, with either a small increase (NH_3_ and H_2_S) or decrease (SO_2_) in R. However, the response is low at environmental pollution levels. The response to NO_2_ also is near-zero for permitted long-term exposure. Our sensor is, therefore, not suited for environmental monitoring, but acceptable environmental pollutant levels do not interfere with the sensor. Above a threshold of ~87 ppb, the response to NO_2_ becomes very strong. This response is presumably due to the doping of CuPc by the strongly oxidising NO_2_, and is far stronger than for previously reported CuPc chemiresistors. We relate this to differences in the film morphology. Under 1 ppm NO_2_, R drops by a factor of 870 vs. non-polluted air. An amount of 1 ppm NO_2_ is far above the ‘background’ environmental pollution, thereby avoiding false alarms, but far below immediately life-threatening levels, thus giving time to evacuate. Our sensor is destined for leak detection in the nitrogen fertiliser industry, where NO_2_ is an important intermediate.

## 1. Introduction

The pollution of our atmosphere with toxic gases, such as NO_2_, NH_3_, H_2_S, and SO_2_, is an unfortunate side effect of industrialisation and traffic in the modern world, particularly in the urban environment, and may be particularly intense in some workplaces. For example, NO_2_ is generated by high-temperature combustion in air (diesel engines and gas cookers), but it is also an intermediate in the synthesis of nitric acid, which is produced on a large scale in the fertiliser industry. NO_2_ is, therefore, a common urban and indoor air pollutant causing serious health effects by long-term exposure to lower concentrations, but also poses an imminent danger to life from leaks in fertiliser plants, with a ‘median lethal dose’ LC_50_ for humans of 174 parts per million (ppm) for 1 h. All major legislations therefore set ‘limiting values’ (LVs) or ‘maximum exposure limits’ (MELs) for NO_2_ and other toxic gases. These differentiate between environmental air quality and the permitted exposure of individuals in the workplace, and are typically graded according to the duration of exposure. For example, the EU sets standards for environmental air quality of 200 μg/m^3^ = 106 ppb NO_2_ for a maximum of 1 h, and 40 μg/m^3^ = 21 ppb for ‘1 year’, i.e., permanent exposure [1]. Compliance of actual environmental pollution levels with these targets is comprehensively monitored over large areas by the ‘Copernicus’ Atmosphere Monitoring Service [2], which combines ground-based and satellite monitoring data. Different regulatory regimes apply ‘on the spot’ for industries processing harmful substances. For the workplace, the EU Occupational Safety and Health Agency (OSHA) defines LVs for CO, SO_2_, H_2_S, NH_3_, NO_2_, and NO in Commission directive 2017/164 [3]; values are tabulated in Supplementary Materials, Section S1. US legislation sets short-term ‘emergency exposure limits’ (EELs) to NO_2_ in the workplace between a ‘5-min EEL’ of 35 ppm and a 60-min EEL of 10 ppm [4].

The common pollution of air with the aforementioned four gases (as well as others) calls for sensor technologies for their identification and quantification. Sensors require a ‘sensitiser’, i.e., a material that binds to the target analyte and changes (at least) one of its physical properties in response, and a ‘transducer’ that measures the change in this physical quantity. A family of materials that are known to bind and respond to toxic and chemically aggressive gases are organic dyes and semiconductors, i.e., organic materials with extended π-conjugated electronic orbitals. Air- and waterborne pollutants often interact with organic dyes and semiconductors, and change their optical (e.g., [5]) and/or electronic properties, making them potential sensitisers for such pollutants. Historically, organic semiconductor-based sensors were often transduced by organic field-effect transistors, known as OFETs or OTFTs, which change their electronic characteristics in line with the semiconductor’s electronic properties. When the OFET is actuated by a gate voltage, the field effect enhances the charge carrier concentration in the transistor’s channel, and, therefore, its conductivity, by many orders of magnitude. This is helpful for measurement, as the charge carrier mobility in organic semiconductors is often low. Also, OFET characteristics may reveal other parameters than the carrier mobility, in particular, the threshold voltage, which may also be employed for the transduction of airborne gases. Examples for real-time and/or multiparametric OFET characterisation systems for gas sensing are, e.g., found in [6,7]. The poly(triaryl amine) (PTAA) organic semiconductor family has proved to be particularly sensitive to NO_2_, leading to solution-processed OFET-based NO_2_ sensors, as reported by Das et al. [8]. Cui et al. [9] later reported a solution-processed copper phthalocyanine (CuPc) OFET sensor for NO_2_, but the poor solubility of CuPc demands a toxic perfluorinated organic acid as a solvent, resulting in transistors that show very small saturated drain currents even at high gate voltages (~100 nA at 60 V), and the reported limit of detection of 300 ppb is not particularly low. Anisimov et al. [10] designed and tested a vacuum-evaporated OFET array for the analysis of ambient air with respect to the identity and quantity of air pollutants, such as NO_2_, NH_3_, H_2_S, and SO_2_, based on OFETs using different metalloporphyrins (Cu/Zn/TiO–porphyrin) as organic semiconductors. A review of OFET gas sensors was performed, e.g., by Trul et al. [11].

As impressive as some of these works are, the preparation and electric characterisation of OFET sensor devices require formidable skills and instrumentation. A far simpler approach to gas detection is based on ‘chemiresistors’, i.e., devices that are (only) characterised by their electric resistance while exposed to potentially contaminated air. Such chemiresistor sensors have been realised by common insulating polymer matrices filled with conductive ‘carbon black’ (CB) particles. The response to airborne pollutants is not by a direct impact on the conductivity of CB, but by the somewhat selective swelling of different polymer matrices when exposed to airborne pollutants, and the consequential increase in the separation of CB particles leading to reduced conductivity. The only moderate selectivity of swelling calls for the use of sensor arrays with a wide range of polymer matrices, and the subsequent analysis of the array’s response pattern with sophisticated methods. The historic ‘breakthrough’ contribution to this field was the work of Lewis et al. [12]. Such arrays are very useful for the identification and quantification of vapours rather than gases, i.e., molecules escaping into the headspace above a liquid that is thermodynamically stable at ambient pressure and temperature. Typical target analytes are vapours of saturated, unsaturated, and aromatic hydrocarbons, chlorinated alkanes, alcohols, and ketones. A recent review was performed by [13].

However, air pollutant gases like NO_2_, NH_3_, H_2_S, and SO_2_ are harmful at concentrations that are too low to elicit a strong swelling response, and are better sensed by materials that respond directly to their electronic transport properties, like the organic semiconductors in OFET gas sensors introduced above. A summary of a few studies using metalophthalocyanines (MPcs) in electronic gas sensors is shown in Table 1.

We note the vapour giving strongest response depends not only on the respective metal in the MPc, but also on the transducer type and the processing details. For example, Li et al. [15] found that vacuum-deposited CuPc transduced with a field-effect transistor responded to sulphur-containing vapours, while solution-processed CuPc transistors responded to NO_2_ and NH_3_ [8].

Thanks to the progress of bespoke integrated circuit (IC) voltage followers, the measurement of very high resistances has recently become practically viable. Special engineering methods raise the input resistance of the commercially available voltage buffer Analog Devices AD8244 to an ultra-high value of 10 TΩ [18]. This brings the measurement of resistances up to at least 100 GΩ into range with a small experimental footprint. The lower device resistance that is achieved in transistors has, therefore, become less of an advantage over the chemiresistors, which are easier to manufacture. Therefore, here we study chemiresistor gas sensors based on copper(II)phthalocyanine, CuPc, a close chemical relative to the metalloporphyrins used by Anisimov et al. [10]. Beyond its widespread use as blue pigment, CuPc has been used as a hole-transporting organic semiconductor, e.g., in organic solar cells [19]. We find that CuPc chemiresistors respond to a variety of harmful gases, including NH_3_, H_2_S, and SO_2_, at low-to-moderate concentrations. In some cases, the response is ‘positive’, i.e., the chemiresistor resistance R increases under exposure. We find a ‘negative’ response, i.e., reduced resistance R, only under NO_2_ and SO_2_, making our sensor selective for different types of gases. While these findings qualitatively agree with a prior report by Chia et al. [14], we find significant differences in the quantitative response to NO_2_. Our chemiresistor shows almost no response to NO_2_ in the concentration range of permitted environmental LVs (<100 ppb), but responds very strongly above a threshold concentration of 87 ppb. This characteristic destines our sensor for workplace applications where there is a risk of NO_2_ leakage.

## 2. Experimental Section

### 2.1. Chemiresistor Preparation and Characterisation

**Figure 1:**
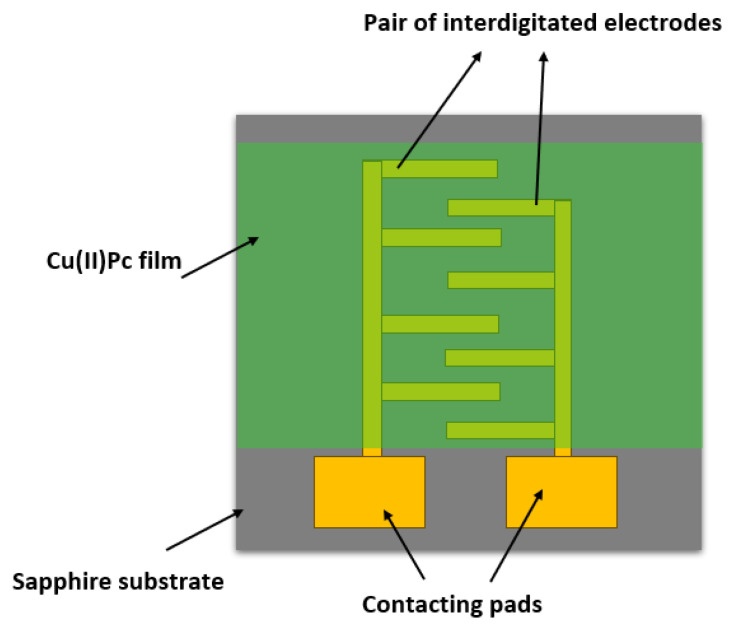
Schematic illustration of the Cu(II)Pc chemiresistor with an interdigitated electrode geometry. For clarity, the illustration is not to scale, and shows fewer than n = 15 interdigitated electrode pairs.

Copper(II)phthalocyanine, CuPc, was sourced from Sigma-Aldrich (Cat No. 459712), and was thermally evaporated onto a sapphire (Al_2_O_3_) supporting substrate in a high vacuum thermal evaporator (E306A, Edwards Vacuum, Crawley, England). The substrate carried interdigitated Ti/Au electrodes (n = 15 pairs with a L = 50 μm channel length and a W = 1 mm overlap, as well as a geometry factor n W/L = 15 × 1 mm/50 μm = 300). The electrode geometry is illustrated schematically in Figure 1.

We loaded 5 mg to 10 mg of CuPc into a quartz boat mounted in a tungsten heater filament and started the deposition process after the vacuum base pressure had dropped to ~1 × 10⁻⁵ mbar. Initially, we gradually increased the heater current while keeping the shutter closed and monitored the vacuum. A slight increase in the base pressure marked the start of evaporation; first, we allowed a few minutes of evaporation with a closed shutter for the impurities to evaporate, and then opened the shutter. Deposition occurred in two stages. First, low-rate deposition, evidenced by minor vacuum fluctuations and the gradual increase in the opacity of the bell jar. Second, we followed this with a high-rate deposition (~1–5 nm/s) by increasing the heater current until the complete evaporation of CuPc from the boat resulting in a dark blue film on the sapphire substrate. The deposition rate was estimated from the time duration and final thickness, cf. Section 3.1.

The large geometry factor somewhat compensated for the high resistivity of the CuPc films. Subsequently, the film thickness was determined using a Dektak surface profilometer, and the film surface was imaged using a Bruker Dimension Icon AFM with a FastScan A tip and ScanAsyst mode. We then used ImageJ software (1.51j8) [20] to analyse the grain size and surface roughness.

### 2.2. Generation of Pollutant Atmospheres for Calibration

Gases were bought in cylinders from AHG (Saudi Arabia) with supplier-certified gas concentrations, as follows: H_2_S, 20 ppm; NO_2_, 10 ppm; SO_2_, 9 ppm; NH_3_, 200 ppm. The gases were diluted using compressed high-purity dry ‘zero’ air with the help of a GSM 3000 gas sensor measurement system [21], which is made of four mass flow controllers (MFCs) and a static mixer so to ensure a complete mixing of two gases or the dilution of a gas with air. We checked the nominal gas flow rates delivered by the GSM 3000 against an external MFC, and found that both readings matched within a very narrow range. We express analyte concentrations c in dimensionless form as partial pressure, p/p_atmosphere_, which according to the ideal gas equation equals the relative abundance. Typical c values are in the range from 10^−9^ (parts per billion, ppb) to 10^−6^ (parts per million, ppm).

### 2.3. Resistance Measurement and Quantification of Sensor Response

The resistance of our chemiresistors was monitored in real time under exposure to defined pollutant atmospheres using a high-resistance meter (Keithley 6517B) with an input impedance of 200 TΩ. To have a measurable resistance, the input voltage was increased to (200 to 500) V. Triaxial cables were used to connect our sensor (inside the chamber) to the High and LO Keithley 6517B inputs. The connection of Keithley’s internal ammeter and the LO of the voltage source was activated by the instrument’s software in the ‘Meter-Connect setting’. The sensor chamber and Keithley instrument were jointly grounded to avoid any parasitic effects. The film resistance prior to the gas exposure was in the order (400 to 900) MΩ. For the evaluation, data are presented as a graph resistance R vs. time t, R(t). The times at which the gas exposure begins or the gas concentration changes are indicated on the t axis. R(t) may drift somewhat even under zero exposure, and does not always immediately respond fully to gas exposure, but for a given gas concentration c, it approaches a limiting value with a response time constant τ. We define a dimensionless response r(c) as this limiting resistance value at a given concentration c of a particular gas, divided by the resistance prior to exposure, as shown in Equation (1):

r(c) = R(c, t >> τ)/R(c = 0)(1)

Normalising the response r(c) to the initial resistance R(c = 0) accounts for the variability in the device manufacture. To quantify the response time constant τ, we sometimes fit the approach to the full response over time to an exponential law, as follows:

R(c, t) = R(c, t = 0) + ΔR(c, t → ∞)exp(−t/τ)(2)

where τ and ΔR(c, t → ∞) are the fit parameters, and t = 0 is defined by the onset of gas exposure. Equation (2) also allows for the extrapolation of R(c, t >> τ) for Equation (1) as R(c, t = 0) + ΔR(c, t → ∞) in cases where τ is long and the exposure was ended before reaching the full response. We call the response ‘positive’ for positive ΔR(c, t → ∞), i.e., r(c > 0) > 1, ‘zero’ for ΔR(c, t → ∞) = 0, i.e., r (c > 0) = 1, and ‘negative’ for a negative ΔR(c, t → ∞), i.e., r(c > 0) < 1.

We define the ‘magnitude’ m of a response by a logarithmic metric, as shown in Equation (3):

m(c) = |log r(c)|(3)

e.g., we would consider the magnitude of r(c) = 1 as zero, since the resistance has not changed, and the magnitudes of both r(c) = 10 and r(c) = 0.1 are equal to 1, and, therefore, equal to each other. This is because it is an arbitrary choice to evaluate the resistance rather than the conductance, in which case all of the responses would be inverted. The magnitude metric in Equation (3) is indifferent to this arbitrary choice.

## 3. Results and Discussion

### 3.1. CuPc Film Morphology

**Figure 2:**
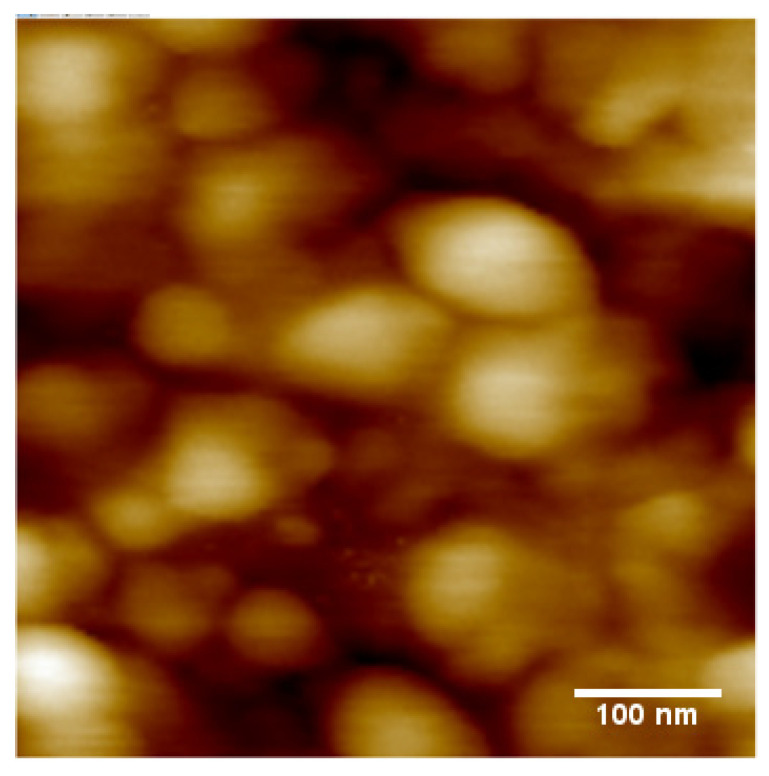
AFM image of the CuPc films deposited on the sapphire substrate showing large-size grains.

The Dektak surface profilometer characterisation of the CuPc films, prepared as described in Section 2.1, returns an average film thickness of ~1 μm and an rms surface roughness of 90 nm, i.e., ~9% of film thickness. An AFM image of the film surface is shown in Figure 2.

The analysis of Figure 2 with ImageJ software returns an average grain size of about 140 nm. Our CuPc deposition conditions, and the resulting morphology, show a number of differences to the evaporated CuPc films studied by Chia et al. [14]. They used a silicon/silicon oxide substrate rather than sapphire (aluminium oxide), and a constant rather than increasing deposition rate. Their final film thickness was only (100 … 200) nm, and their films had a significantly smoother surface, with an rms roughness of only vs. (3 … 4) nm. We also obtain significantly larger grain sizes. Chia et al. [14] did not report on the grain size, but, when we analyse their Figure 2 with ImageJ software, we find an average grain size of 44 nm/53 nm for their films of a 100 nm/200 nm thickness.

### 3.2. CuPc Chemiresistor Response to H_2_S and NH_3_

We first tested CuPc chemiresistors under the harmful gases H_2_S and NH_3_ at concentrations on the order of 1 ppm, which represents typical environmental pollution levels.

**Figure 3:**
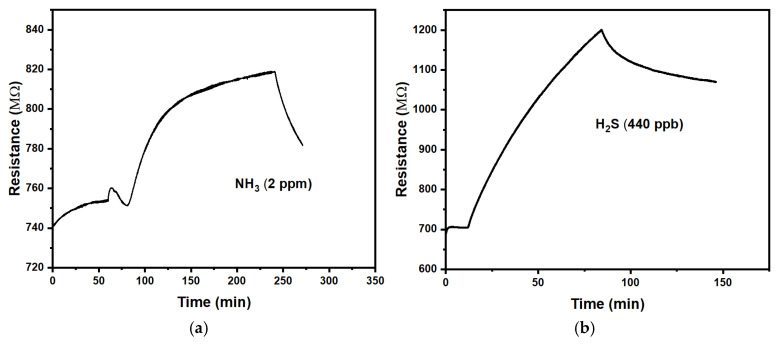
Exposure of the CuPc chemiresistors to (**a**): ammonia, NH_3_; (**b**): hydrogen sulphide, H_2_S.

Figure 3 shows a positive response (r > 1) of the CuPc chemiresistors to the studied gases. Table 2 provides a summary of the quantitative evaluation.

**Figure 4:**
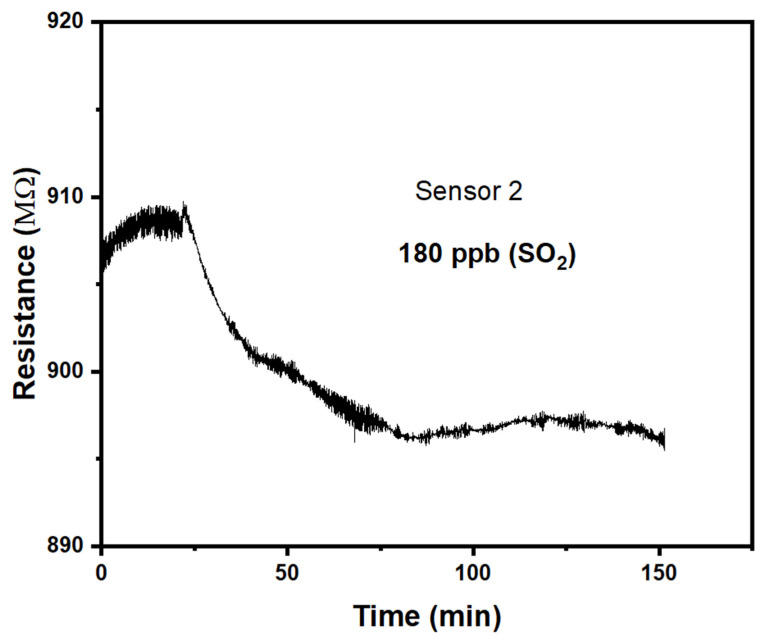
Response of the CuPc chemiresistor to 180 ppb SO_2_.

We find that the response is slow with time constants in the order of (0.5 to 1) h. Recovery after the end of exposure is similarly slow but was not quantified in detail. The magnitude of the responses is relatively small, making it difficult to distinguish a response to a gas from long-term drift in the absence of an analyte gas. Some of this drift is evident from Figure 3. CuPc chemiresistors are, therefore, not well suited for the detection of ammonia and hydrogen sulphide. Precise comparisons to the CuPc chemiresistors reported by Chia et al. [14] are difficult because they did not wait for, or extrapolate to, the full response under a given analyte concentration. However, a rough estimate for the response of their device to 2 ppm NH_3_ can be made from Chia et al.’s Figure 4a [14]. They also found some drift in the absence of analyte odour and a positive response (increased resistance) under exposure, with a faster τ (~2 min) but a smaller magnitude (m ~ 0.03). The faster response is likely due to their use of thinner films.

The small magnitude of the responses to NH_3_ and H_2_S largely disqualify our chemiresistors as sensors for these pollutants. However, this also means that NH_3_ and H_2_S do not act as strong interferants for the detection of our main ‘target’, NO_2_.

### 3.3. CuPc Chemiresistor Response to SO_2_ and NO_2_

We find that the response to SO_2_ and NO_2_ differs qualitatively from the response to H_2_S and NH_3_, studied previously. Namely, the response is now ‘negative’, r < 1, which makes the CuPc chemiresistors selective between (H_2_S or NH_3_) vs. (SO_2_ or NO_2_.) Figure 4 shows the response of a CuPc chemiresistor exposed to a 180 ppb SO_2_ atmosphere. This is significantly larger than the 24 h average air quality standard LV in the EU of 125 μg/m^3^ = 44 ppb [1].

The response is now clearly ‘negative’, i.e., r < 1, albeit the response is only somewhat larger than the noise levels, and is again slow with time. Fitting the approach with time to ΔR(t →∞) with Equation (2) fits less well than previously, shown in Figure 3a,b, but still allows for an estimate of τ = 24 min. We find a response of r(180 ppb) = 0.983, i.e., a response magnitude of m = 0.0074. While this is clearly qualitatively different from previous results, in the sense of a ‘negative’ (r < 1) rather than a ‘positive’ (r > 1) response, the response of the CuPc chemiresistor is both too slow and of too small a magnitude for its use as a sensor for harmful levels of SO_2_ pollution. On the other hand, typical environmental levels of SO_2_ pollution will not interfere with or deliver false positives for the detection of other pollutants.

**Figure 5:**
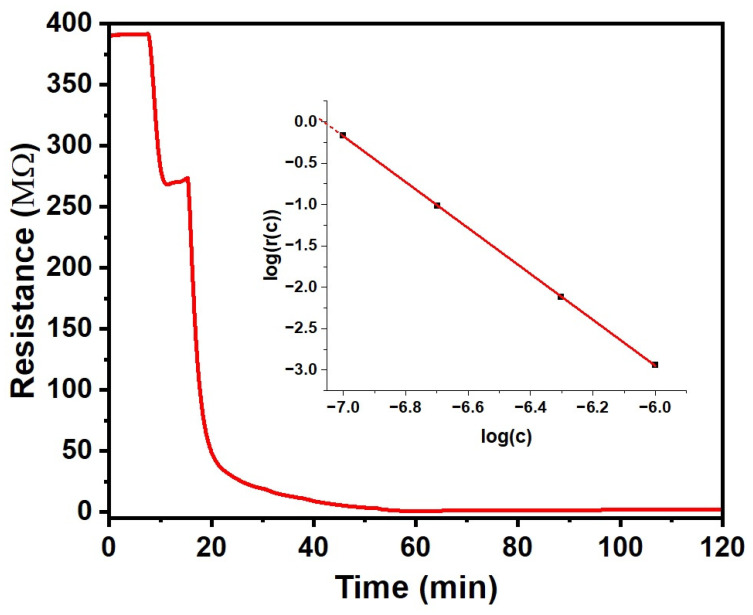
Response of the CuPc chemiresistor to NO_2_. (**Inset**): Response r(c) shown in a logarithmic plot, log r(c) = −m, showing a power law between r and c. The red line is given by Equation (4a).

We find a much stronger ‘negative’ response to NO_2_, which we have, therefore, studied under a range of concentrations from 100 ppb to 1 ppm; the results are shown in Figure 5.

We find a strong negative response (r << 1) under exposure to NO_2_ concentrations of 100 ppb and more. The reduced resistance is most likely due to the oxidative doping of CuPc by the strong oxidising agent NO_2_, making far more charge carriers available for conduction. The response also develops significantly quicker than for the vapours reported in Section 3.2, making a fit to an exponential approach, as in Equation (2), unnecessary to calculate the response r(c) with Equation (1). The inset to Figure 5 shows the response magnitude m = |log r(c)|r(c) against a logarithmic c scale. For c = 100 ppb and above, m vs. log c is fitted well by a straight line, as given in Equation (4a). Extrapolating to below c = 100 ppb (log c = −7) suggests zero response, i.e., r(c) = 1, for log c = −7.06, i.e., c = 87 ppb. Since r(c) = 1 for c = 0 as well by definition, the CuPc chemiresistor does not respond for NO_2_ concentrations below and up to ~87 ppb. Therefore, we take the 87 ppb threshold as a limit of detection (LoD). Our sensor is, therefore, ‘blind’ to environmental ‘background’ NO_2_ levels from common pollution sources, which can be seen as an interferant in the detection of NO_2_ leaks. However, for NO_2_ concentrations c = 100 ppb and more, we find a strong response that is well described by a power law with an exponent close to the inverse third power in c. For c = 1 ppm (log c = −6), the resistance drops by 870-fold, i.e., the magnitude of the response at 1 ppm, in the sense of Equation (3), equals 2.94. This far exceeds the magnitudes observed for the ‘positive’ responses in Section 3.2, and far exceeds any drift. The line in the inset Figure 5 is given by Equation (4a), wherein the response magnitude m is defined as in Equation (3). Equation (4a) may be inverted into Equation (4b) to calculate the NO_2_ concentration c from the measured r(c) for c > 87 ppb, as follows:

m = −log r(c) = 2.774402 log c + 19.59403 (c > 87 ppb)(4a)

c = 87 ppb (1/r)**^0.36043^** (c > 87 ppb) (4b)

Although both we and Chia et al. [14] used vacuum-evaporated CuPc chemiresistors for the detection of NO_2_ (and other gases), we find remarkable differences in the observed response characteristics. Similarly, as also observed by Chia et al. [14], we find that the responses to NH_3_ and NO_2_ are distinguished by their different signs, with NH_3_ leading to an increase and NO_2_ leading to a decrease in the film resistance. We assign the drop in Cu(II)Pc resistance under NO_2_ to oxidative doping by the strongly oxidising NO_2_ radical. Cu(II)Pc becomes more conductive when it is oxidised, i.e., loses electrons. Chemical oxidation is akin to the application of a negative gate voltage to a Cu(II)Pc field-effect transistor, which also turns the transistor ‘on’ into a more conductive state, as reported, e.g., by Cui et al. [9].

However, the response characteristics to NO_2_ display remarkable differences. Qualitatively, the ‘threshold’ behaviour observed here is, in fact, also hinted at in the data shown by Chia et al. [14] in their Figure 3b, with a slightly smaller threshold. However, it is not recognised as such, since the response at a low c was not investigated. Quantitatively, however, above threshold, we observe a response that is orders-of-magnitude larger than that reported by Chia et al. [14]. Their response characteristics are fitted linearly to an increasing c with a rather shallow slope, while we find a power law with an exponent of 2.77. For example, the magnitude of their response at 500 ppb NO_2_, in the sense of our Equation (3), is only m = 0.0645, while we find a response with a magnitude m = 2.12. We note that our faster deposition rate in the latter stages of evaporation has led to rougher morphologies (cf. Section 2.1 and Section 3.1), which is in agreement with the observations of Das et al. [22]. It is widely known that the granular morphology of organic semiconductors strongly influences the current/voltage characteristics, mostly via the properties of junctions at grain boundaries. Detailed reviews are, e.g., found in Stingelin et al. [23] and Bi et al. [24]. Oxidative doping strongly increases a carrier’s ability to tunnel across grain boundaries, similarly as at a Schottky junction. We note that the Schottky junction current is strongly non-linear with the doping level, so the observed non-linear response to the NO_2_ concentration (i.e., the doping level) here is no surprise. Our morphology appears to emphasise the importance of grain boundaries much more so than in the smoother films prepared by Chia et al. [14]. Our rougher surface may also facilitate the better ingress of analyte vapour into the bulk film. We cannot provide a full understanding here, which would require deeper morphology investigations (e.g., cross-sectional SEM or X-ray diffraction) that are beyond our capabilities. However, empirically, our response characteristics are well adapted for the detection of NO_2_ leaks in industrial settings.

### 3.4. Recovery and Resilience

In Section 3.2 and Section 3.3, we report the strong response of our sensor to high levels of its target analyte, NO_2_, with clear selectivity over both low (environmental) levels of NO_2_ and other harmful pollutants. While this establishes the potential of our device as an industrial NO_2_ leak sensor, these results have been obtained under laboratory conditions. A practical sensor needs to be certified under more realistic conditions. We explore some of these in the following, without claiming to be comprehensive.

Following the exposure to 1 ppm NO_2_, our sensor recovers slowly when purging with clean air. Full recovery can take several hours, at least two orders of magnitude slower than the response. This is in contrast to the recovery from the exposure to NH_3_ and H_2_S, which was similarly quick as the response (cf. Figure 3). It is typical for sensors with a large response magnitude to only recover slowly, and accelerated recovery would require heating. Slow recovery is another difference to the characteristics observed by Chia et al. [15]. Their recovery after NO_2_ exposure was (only) ~two times slower than the response, and not two orders of magnitude slower, as with our films; however, their response to NO_2_ was significantly weaker.

When we repeatedly exposed/recovered the sensor used for Figure 5 over prolonged periods, it displayed signs of ageing, with the initial resistance increasing to >1 GΩ. Ageing will, therefore, not be mistaken for an NO_2_ leak, which instead leads to a large drop in resistance. Also, aged chemiresistors still show a significant drop of resistance under 1 ppm NO_2_ exposure, although not by a factor 870. Chemiresistors used and recovered repeatedly over ~1 year and, when stored under ambient conditions for 6 months, still responded with a resistance drop by a factor ~10 under 1 ppm NO_2_. This is still a significantly larger response than previous reports, even on pristine Cu(II)Pc chemiresistors [14], which do not report longevity studies under ambient storage. We do not see ageing after exposure/recovery as a major disadvantage. NO_2_ leaks to 1 ppm + will hopefully be rare events, and chemiresistor preparation is rather simple, i.e., cheap. We recommend that, once exposed to ‘alarm’ levels, the sensors shall be exchanged for pristine replacements. Similarly, sensors shall be replaced even without ‘alarm’ events at regular intervals (~1 year).

For our response characterisation, the Cu(II)Pc chemiresistors were thermostatted at 20 °C in entirely dry air. Thermostatting and humidity control are unrealistic in practical settings, and would require further testing before the practical deployment of our sensors. However, we do not believe that the sensors will fail under typical atmospheric conditions. Cui et al. [9] even found an increase in the Cu(II)Pc sensor response to NO_2_ with increasing relative humidities (RHs) under typical atmospheric values of (35 to 75)% RH. When thermostatting our sensor to 40 °C, we still find a manifold response under 1 ppm NO_2_, albeit with a response magnitude that is about halved (see Figure S1 in the Supplementary Material). We did not yet test the sensors under ‘cross’-exposure, e.g., NH_3_ and NO_2_ simultaneously, as this is unlikely to happen in a NO_2_ processing industrial unit. There will be no other harmful gases processed in parallel. Even in such a case, the much stronger response to NO_2_ than to any other gases is unlikely to be masked by their parallel presence.

## 4. Conclusions

Here, we present a selective chemiresistor sensor for NO_2_ based on a thin film of the organometallic semiconductor copper(II)phthalocyanine (CuPc). The sensor is easily manufactured, and can be read by a simple resistance (R) measurement. The R is high but within the measurement range of modern voltage buffer ICs like the AD8244 [18]. The CuPc film shows small-to-moderate response under exposure to several harmful gases. CuPc responds with an increase in the resistance (‘positive’ response) to NH_3_ and H_2_S, and with a negative response to SO_2_ and NO_2_. This gives our sensor some selectivity, as the sign of the response allows us to discriminate between gases. The response to SO_2_ is very low up to 180 ppb, r(180 ppb) = 0.983, magnitude m = 0.0074, when 180 ppb is already significantly larger than the permitted environmental SO_2_ pollution levels. Common environmental pollutants will, therefore, not interfere with the NO_2_ detection. The sensitivity to NO_2_ also is low or zero in the range of permitted long-term or permanent exposure limits, with a limit of detection (LoD) of ~87 ppb vs. the EU permanent exposure limit of 21 ppb [1]. Our sensor is, therefore, not well suited for environmental monitoring. However, 87 ppb NO_2_ acts as a threshold, above which the sensitivity becomes very high, presumably due to the doping of CuPc by the strongly oxidising NO_2_. Our sensor is, therefore, destined for industrial settings, particularly in the nitrogen fertiliser industry, where NO_2_ leakage to concentrations far above typical environmental pollution levels poses a clear and present danger. The response characteristic of our sensor is well adapted to warn of such leaks. There is a near-zero response to typical and sometimes acceptable environmental ‘background’ NO_2_ levels <87 ppb. However, in the case of a NO_2_ leak, there will be a ‘shrill’ warning, e.g., at 1 ppm NO_2_, the film resistance R is reduced ~ 870 times (response magnitude m = 2.94) vs. the un-exposed sensor within 50 min. While 1 ppm NO_2_ is far above the ‘background’ environmental pollution, it is still well below immediately life-threatening levels, e.g., the short-term NO_2_ ‘emergency exposure limit’ (EEL) is 10 ppm for 1 h [4]. Therefore, our sensor gives a clear warning when there still is time for staff to evacuate.

The responses to NO_2_ reported here for our chemiresistors are significantly larger than in the previous report by Chia et al. [14], despite using the same organometallic semiconductor, CuPc. We note a few significant differences in the preparation of semiconducting films, with consequential differences in the morphologies between our films and those reported by Chia et al., [14] cf. Section 2.1 and Section 3.1. While we cannot give a detailed ‘cause and effect’ explanation for the much enhanced response observed by us, we conclude that our morphology enhances the chemiresistor response.

Overall, we here report a chemiresistor for NO_2_ with a pronounced ‘threshold’ behaviour. We find no response at environmental ‘background’ levels, but a remarkably strong response at immediately harmful levels. Studies on sensor ageing and the response to interferants suggest such chemiresistors may perform as industrial NO_2_ leak detectors under real-world conditions. We encourage more detailed interference and ageing studies by industrial sensor developers for full certification.

## Figures and Tables

**Table 1:** Some previous studies using metalophthalocyanines as sensitisers for harmful gases in electronic sensors. The analyte vapours giving the strongest responses are shown. Sometimes, the analytes can be distinguished by the response sign.

MPc	Transducer	Analyte Vapour(s)	Ref.
CuPc	Transistor	NO_2_	[9]
CuPc	Chemiresistor	NO_2_, NH_3_	[14]
CuPc	Transistor	H_2_S, SO_2_	[15]
ZnPc	Chemiresistor	NO_2_, NH_3_	[16]
TiOPc	Chemiresistor	NO_2_	[17]
CuPc	Chemiresistor	NO_2_	Here

**Table 2:** Summary of the response characteristics for the CuPc chemiresistors under H_2_S and NH_3_. The time constant τ and response magnitude m are defined in Equations (2) and (3), respectively.

Gas	c [ppm]	τ [min]	M
H_2_S	0.44	63.1	0.31
NH_3_	2	28.2	0.03

## Data Availability

The raw data supporting the conclusions of this article will be made available by the authors on request.

## Supplementary Materials

The following supporting information can be downloaded at: https://www.mdpi.com/article/10.3390/s25092955/s1, Figure S1: The response and recovery of a 1-year-old Cu-Pc film for 1 ppm of NO_2_/zero air at different temperatures: (a) Response and recovery behaviour at 20 °C. The plot shows the change in resistance during NO_2_ exposure, followed by the recovery phase in zero air, illustrating the sensor’s sensitivity and recovery speed at ambient temperature. (b) Response and recovery behaviour at 40 °C under the same conditions. The increased temperature results in a similar response magnitude but a notably faster recovery, indicating that higher temperature facilitates desorption and sensor reset; Table S1: OSHA limit values (LVs) for short term or prolonged (8 hr) exposure to several toxic gases. Toxic gas concentrations are expressed as partial pressures in parts-per-million (ppm) of atmospheric pressure, p/patm [3].
