# Supplementary material for: Copper Phthalocyanine Chemiresistors as Industrial NO2 Alarms
Source: Sensors (Basel). 2025 May 7;25(9):2955. doi: 10.3390/s25092955 (PMC12074087; doi:10.3390/s25092955)
Supplement: Supplementary file 1 [file sensors-25-02955-s001.zip › sensors-3571249-supplementary.pdf]

Supplementary material

S1: EU environmental and workplace (OSHA) limit values for air pollutants

Substance	OSHA limit 8 hrs. [ppm]	OSHA limit short term [ppm]
Carbon monoxide (CO)	20	100
Sulphur dioxide (SO_2)	0.5	1
Hydrogen sulphide (H_2S)	20	50
Ammonia (NH_3)	20	50
Nitrogen dioxide (NO_2)	0.5	1
Nitrogen monoxide (NO)	2	-

Table S1: OSHA limit values (LVs) for short term or prolonged (8 hr) exposure to several toxic gases. Toxic gas concentrations are expressed as partial pressures in parts-per-million (ppm) of atmospheric pressure, p/p_{atm} [1].

[1] *Commission Directive (EU) 2017/164 of 31 January 2017 establishing a fourth list of indicative occupational exposure limit values pursuant to Council Directive 98/24/EC, and amending Commission Directives 91/322/EEC, 2000/39/EC and 2009/161/EU (Text with EEA relevance.)*. Official Journal of the European Union. p. 115-120.

S2: Response and recovery of a 1-year-old Cu-Pc film to 1ppm NO_2 at different temperatures.

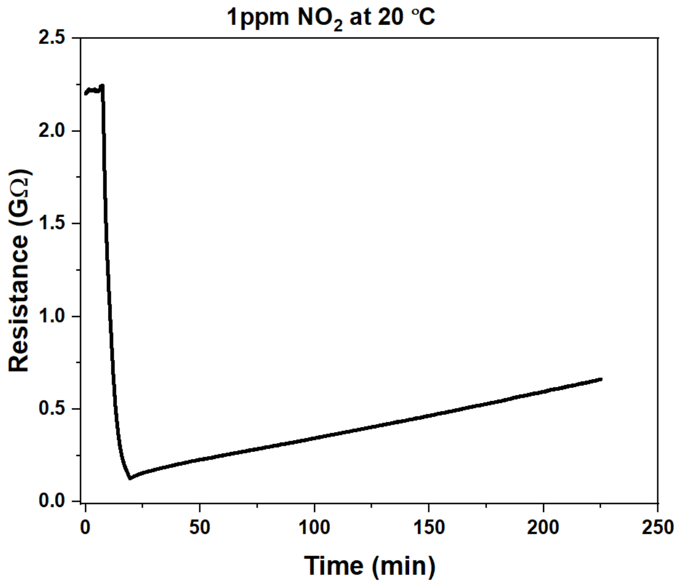

(a)

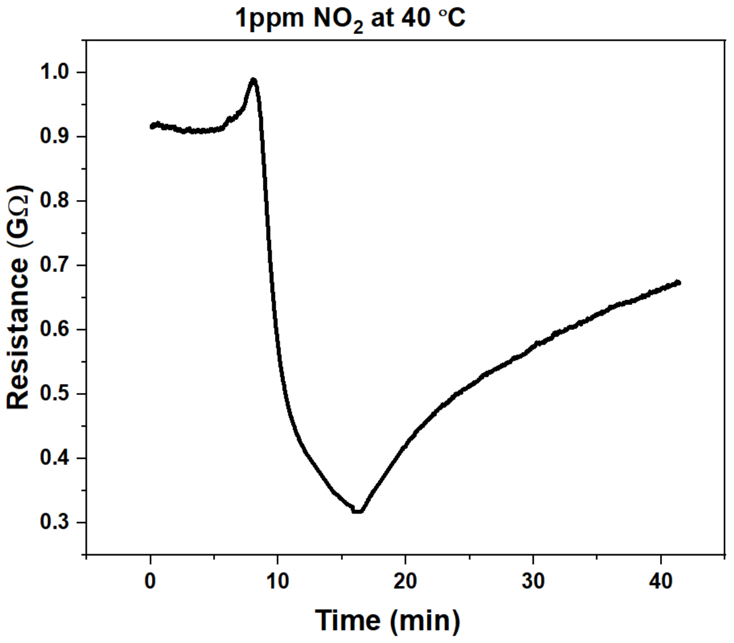

(b)

Figure S1: The response and recovery of a 1-year-old Cu-Pc film for 1 ppm of NO_2/zero air at different temperatures:
(a) Response and recovery behaviour at **20°C**. The plot shows the change in resistance during NO_2 exposure, followed by the recovery phase in zero air, illustrating the sensor's sensitivity and recovery speed at ambient temperature. *(b)* Response and recovery behaviour at **40°C** under the same conditions. The increased temperature results in a similar response magnitude but a notably faster recovery, indicating that higher temperature facilitates desorption and sensor reset.

Note: Both experiments were conducted on the same aged Cu-Pc film stored under ambient conditions for 6 months prior to testing. The data demonstrate that elevated temperature enhances recovery speed even after prolonged storage, supporting the use of gentle heating to restore sensor performance after each exposure session.
